# Immediate prosthetic breast reconstruction after removal of the polyacrylamide hydrogel (PAAG) through a small areolar incision assisted with an endoscope

**DOI:** 10.1186/s12893-022-01778-7

**Published:** 2022-09-07

**Authors:** Dandan Guan, Qiuping Mo, Yajuan Zheng

**Affiliations:** General Surgery, Cancer Center, Department of Breast Surgery, Zhejiang Provincial People’s Hospital (Affiliated People’s Hospital, Hangzhou Medical College), Hangzhou, 310014 Zhejiang China

**Keywords:** Prosthetic breast reconstruction, Polyacrylamide hydrogel, BREAST-Q, Endoscope

## Abstract

**Background:**

To identify the feasibility, safety, cosmetic outcomes and patient satisfaction of immediate prosthetic breast reconstruction after removal of Polyacrylamide Hydrogel (PAAG) through a small areolar incision assisted with an endoscope.

**Methods:**

This was a retrospective study. Medical records of 87 patients who underwent PAAG removal were reviewed retrospectively from February 2010 to December 2019. These patients were dichotomized based on whether they accepted immediate prosthetic breast reconstruction after PAAG removal or not. A comprehensive analysis on the data was conducted to observe the surgical results, cosmetic outcomes, health-related quality of life (HRQOL) and patient satisfaction.

**Results:**

Sixty-two patients underwent PAAG removal through a small areolar incision assisted with an endoscope, while another 25 patients underwent further immediate prosthetic breast reconstruction after PAAG removal. All the patients recovered smoothly after operation. In the immediate breast reconstructed group, most of the breasts were natural in appearance, but one patient had mild nipple and breast asymmetry, and another had mild breast asymmetry. Three patients had PAAG residual, and one of them accepted fine needle aspiration. The cosmetic satisfaction rate was 88% and 92% by surgeons and patients, respectively. In the other group, seven patients suffered from PAAG residual, one patient suffered from postoperative bleeding, and five patients suffered from skin laxity. The BREAST-Q scores revealed that patients who accepted immediate breast reconstruction had significant better outcomes in psychosocial well-being (*p* = 0.030), satisfaction with breasts (*p* = 0.021), when compared to patients who only accepted PAAG removal, while similar in sexual well-being (*p* = 0.081), physical well-being chest (*p* = 0.124), and satisfaction with outcomes (*p* = 0.068), and satisfaction with care (*p* = 0.077).

**Conclusion:**

Immediate prosthetic breast reconstruction after PAAG removal through a small areolar incision aided with an endoscope might be a viable and safe technique, with better psychosocial well-being and satisfaction with breasts.

## Background

Polyacrylamide hydrogel (PAAG) had been widely used for breast augmentation in China for nearly 10 years [1]. It was a polymer synthesized from 2.5% cross-linked polyacrylamide and 97.5% pyrogenic water [2]. It was first imported to China from Ukraine in 1997 [3] and was supposed to be an excellent breast augmentation which was non-irritating, non-toxic, and non-teratogenic [4]. It was roughly estimated that 200,000 Chinese women accepted PAAG injection for breast augmentation [5]. But its safety had been questioned since the complications of PAAG injection were reported in succession [6]. The production and application of PAAG was prohibited by the China Food and Drug Administration (CFDA) in 2006. Currently, complications caused by PAAG injection are still very common and have become a major public health issue. Pain, lumps, breast hardening, deformity, and migration are common complications [7], which may further lead to noticeable physical and mental damage [8].

A large number of PAAG injected patients have continued to seek medical advice to treat the complications. An extensive surgical debridement including PAAG evacuation, pathologic tissue excision and pocket irrigation, was usually in demand to remove the PAAG tissues [9]. Nevertheless, the extensive surgery often led to nipple retraction, postoperative mastoptosis, breast asymmetry, and skin laxity [10, 11].

Breast reconstruction was highly required after PAAG removal to handle the aesthetic problem. It was reported that silicone prosthesis implantation after PAAG evacuation was effective for breast contour restoration for patients who fell under a particular category [12]. It was also said that silicone prosthesis implantation (immediate or delayed) was commonly practiced after PAAG removal [13].

Some experts suggested delayed breast reconstruction when it came to the timing of prosthesis implantation, considering the residual PAAG, and destroyed inframammary fold and pectoralis major [14]. In contrast, some experts supported immediate prosthesis implantation when the indications were strictly controlled [12]. It was also stated that there was no substantial evidence pointing to an increased complication rate after immediate prosthesis implantation [12]. In our clinical practice, PAAG removal was usually executed through a small areolar incision assisted with an endoscope for a relatively complete removal. Prosthesis implantation was performed immediately for selected patients. In this paper, we reviewed our past medical records and reported our experience on the technique of immediate prosthetic breast reconstruction after PAAG removal through a small areolar incision assisted with an endoscope.

## Methods

### Patients

This was a retrospective study. Medical records of 87 patients who underwent PAAG removal through a small areolar incision assisted with an endoscope were reviewed retrospectively from February 2010 to December 2019. Patients who met the following indications accepted further immediate prosthetic breast reconstruction: (1) It was expected that the breast defect or deformation after PAAG removal was obvious; (2) Patients had a strong desire for breast reconstruction; (3) Adequate normal breast tissue was conserved to cover the prostheses; (4) PAAG tissue was removed as completely as possible, and there was no significant residue. The same surgical team performed all the surgical procedures. The Research Ethics Committee of our institution approved this study. Written informed consent forms for the publication of this report and accompanying images were all gathered from the patients.

Preoperative routine examinations were executed in all patients to exclude surgical contraindications. It was common to perform ultrasound and magnetic resonance imaging (MRI) to thoroughly estimate the volume, range and distribution of PAAG tissue before the surgery. Patients were omitted from immediate breast reconstruction if they were diagnosed as breast cancer or acute inflammation in the meantime. Fully preoperative communication and written informed consent was required. The patients must be fully informed of the surgical risks, such as residual PAAG, infection, bleeding, mastoptosis, nipple retraction, capsular contracture, breast or nipple asymmetry, skin laxity, breast malignancy, etc.

A cosmetic evaluation system of breast set up by Kroll [15] was used, including symmetry, shape, ptosis, and scars (Table 1). The cosmetic result was divided into four grades and defined as excellent, good, fair, and poor, according to the four-category Harvard scale [16]. The surgeons and patients assessed the cosmetic outcomes independently during outpatient follow-up. ‘Satisfaction’ would be considered, when the result was evaluated as ‘excellent’ or ‘good’.

**Table 1:** Assessment standards for cosmetic results

Grade	Symmetry	Shape	Ptosis	Scars
Excellent	Symmetrical	Normal	Nature	No contracture
Good	Slightly asymmetrical	Slight deformation	Slightly unnatural	Slight contracture
Fair	Asymmetrical	Deformity	Unnatural	Contracture
Poor	Very asymmetrical	Severe deformity	Very unnatural	Severe contracture

Besides, patients in the two groups had been asked by a feldsher to complete the BREAST-Q (Augmentation Modules) through telephone follow-up recently. The BREAST-Q, as an accepted and validated questionnaire, was effective in quantifying the impact of immediate prosthetic breast reconstruction on health-related quality of life (HRQOL) and patient satisfaction [17]. Data on psychosocial well-being, sexual well-being, and physical well-being chest, satisfaction with breasts, satisfaction with outcomes, satisfaction with care, were all derived from patients to create the database.

### Statistical analysis

Scores were extracted from patients for each of the BREAST-Q’s domains and transformed to a scale of 0–100. A higher value represented a more favorable outcome, according to the BREAST-Q protocol. Descriptive statistics included the mean and standard deviation (SD). A *t* test was used to compare the outcomes between the two groups. Statistical Package for the Social Sciences (SPSS version 26.0) was utilized for statistical analysis.

### Surgical procedures

**Fig. 1:**
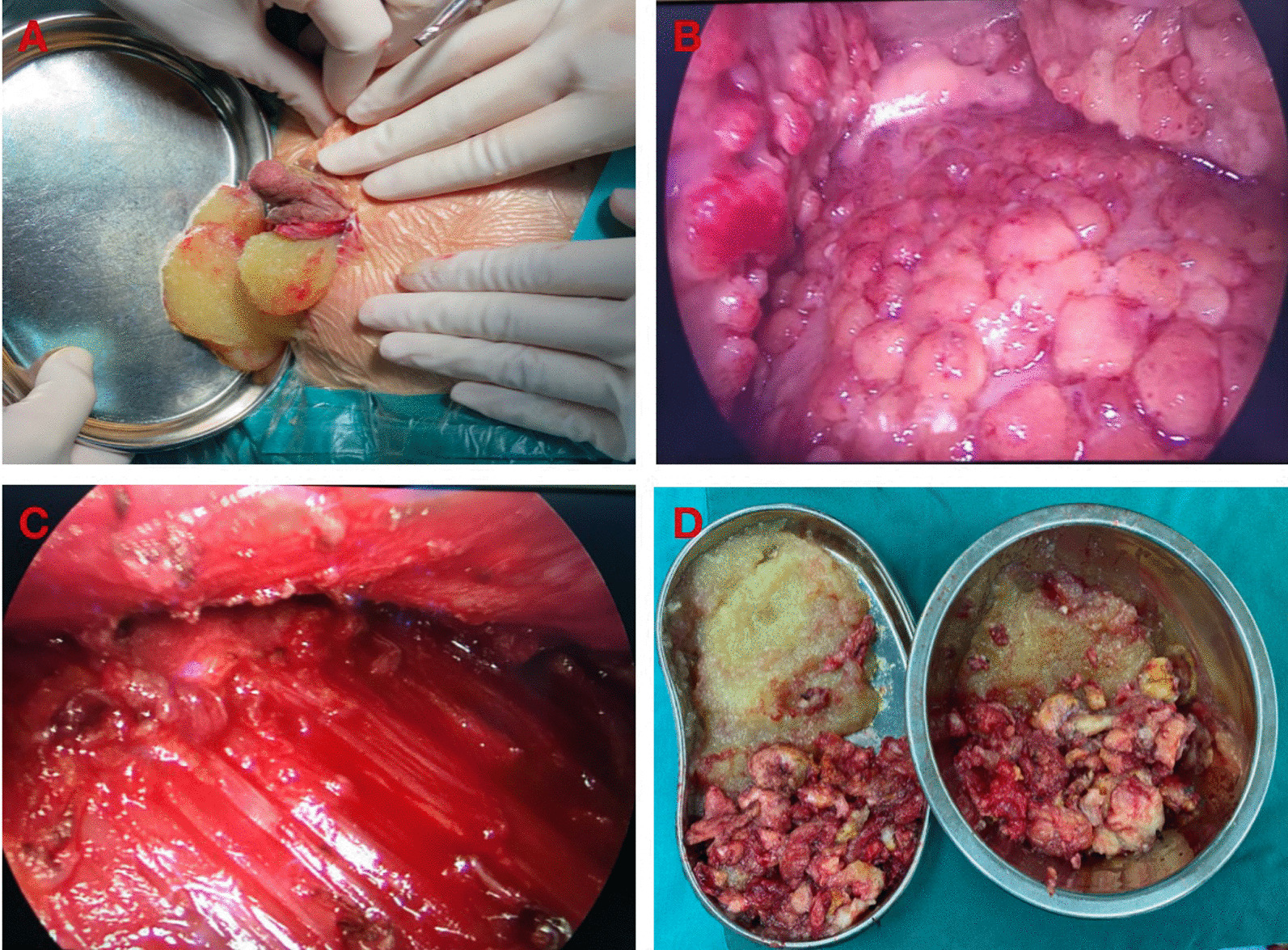
Surgical procedures of PAAG removal. **A** The flowing material was drained after the separation of the capsule. **B** PAAG nodules located in posterior mammary cavity. **C** The posterior mammary cavity after PAAG removal. **D** The removed PAAG tissue, included yellow liquid tissue and nodular tissue

During the surgery, the PAAG removal was carried out through a small areolar incision assisted with an endoscope. Prosthetic breast reconstruction in the pre-pectoral plane was performed immediately for selected cases after PAAG removal. The surgical management was as follows (Fig. 1):

1. Preparations before surgery
  Generally, an MRI was performed to examine the volume and distribution of PAAG tissues in the breasts before the surgery. The surgical incision, middle sternal line, inframammary crest, and scope of surgery were marked in a supine position before surgery. Surgery was carried out in the supine position with bilateral arms resting at 90° abduction after general anesthesia.
2. Removal of PAAG tissue
  An incision about 3–4 cm at the lower edge of the areola was usually chosen. The subcutaneous tissue was dissected to expose the mammary gland. Later, the mammary gland was separated until the capsule of the PAAG in the posterior space was revealed. The flowing material was drained after the separation of the capsule. Most of the liquid PAAG would come out, and the residual breast cavity could be washed fully via a large amount of normal saline.
  A camera (10-mm 30°) was then inserted through the areolar incision to observe the posterior mammary space. PAAG induration, degenerative glandular, and muscular tissues would be observed. Curettage was usually preferred to eradicate the PAAG indurations. It was also recommended to remove the degenerative tissues. The endoscopic instruments were helpful for the assessment and further removal of the residual PAAG and degenerative tissues. Care should be taken to keep the inframammary fold and the pectoral muscle intact. An experienced surgeon was usually in demand to preserve adequate normal mammary gland to cover the prostheses. Besides, additional incisions might be required if the PAAG was significantly displaced outside the breasts, such as supraclavicular or abdominal areas.
3. Prosthesis implantation for immediate breast reconstruction
  Before prosthesis implantation, two breast expanders were used to assess the extent of the defect in bilateral breasts after PAAG removal. Appropriate silicone prostheses were selected for immediate breast reconstruction according to the volume of injected normal saline in the expanders. Before implantation, the prostheses were soaked in the antibiotic solution (cefuroxime or clindamycin) for at least 10 min. Then placed the prostheses in the posterior mammary space and adjusted to a good position. The normal breast tissues could serve as natural coverages of the prostheses. Drainage tubes were placed in the posterior mammary space in bilateral breasts.

## Results

Twenty-five patients underwent immediate prosthetic breast reconstruction after PAAG removal through a small areolar incision assisted with endoscope under strict selection. Sixty-two patients only underwent PAAG removal, and one of them received a delayed prosthetic breast reconstruction. The clinical characteristics, surgical outcomes, and complications of the two groups were listed in Table 2. All patients recovered well after the surgery. The drainage tube was removed when the drainage volume was less than 20 ml per day. It was recommended to wear an elastic garment and massage the breasts for at least 2 weeks after the surgery.

**Table 2:** Patients’ characteristics, surgical outcomes and complications of the two groups

	PAAG removal with immediate prosthetic breast reconstruction	PAAG removal without immediate prosthetic breast reconstruction
Number of patients	25	62
Age (years)	50 (41–58)	47 (33–59)
Interval between injection and debridement (years)	12 (5–20)	13 (3–22)
History of removal, number (%)	2 (8%)	3 (5%)
Operation time (hours)	2.6 (2.0–3.0)	2.0 (1.6–2.5)
Size of prostheses (cc)	180–245	–
Hospital stays (days)	8 (6–10)	6.5 (6–8)
Follow-up time (months)	6–12	6–12
Complications		
PAAG residual	3	7
Nipple asymmetry	1	0
Breast asymmetry	2	0
Postoperative bleeding	0	1
Skin laxity	0	5
Mastoptosis	0	0
Nipple contraction	0	0
Infection	0	0
Capsule contraction	0	–

The patients were followed by outpatient or telephone visits for at least 6 to 12 months. In the immediate breast reconstructed group, three patients had little PAAG residue. Fine needle aspiration was performed in one patient to clear the residue, while the others were under observation. One patient developed mild nipple and breast asymmetry, and another patient had mild breast asymmetry. Both of them refused further breast plastic surgery. Complications such as bleeding, infection, mastoptosis, capsule contraction, or skin laxity were not found. In the other group, seven patients had little PAAG residue, and three of them accepted fine needle aspiration. One patient suffered from postoperative bleeding which was effectively stopped by compression bandage fortunately. Five patients suffered from skin laxity, and one of them accepted a delayed prosthetic breast reconstruction while the others didn’t accept special treatments.

**Fig. 2:**
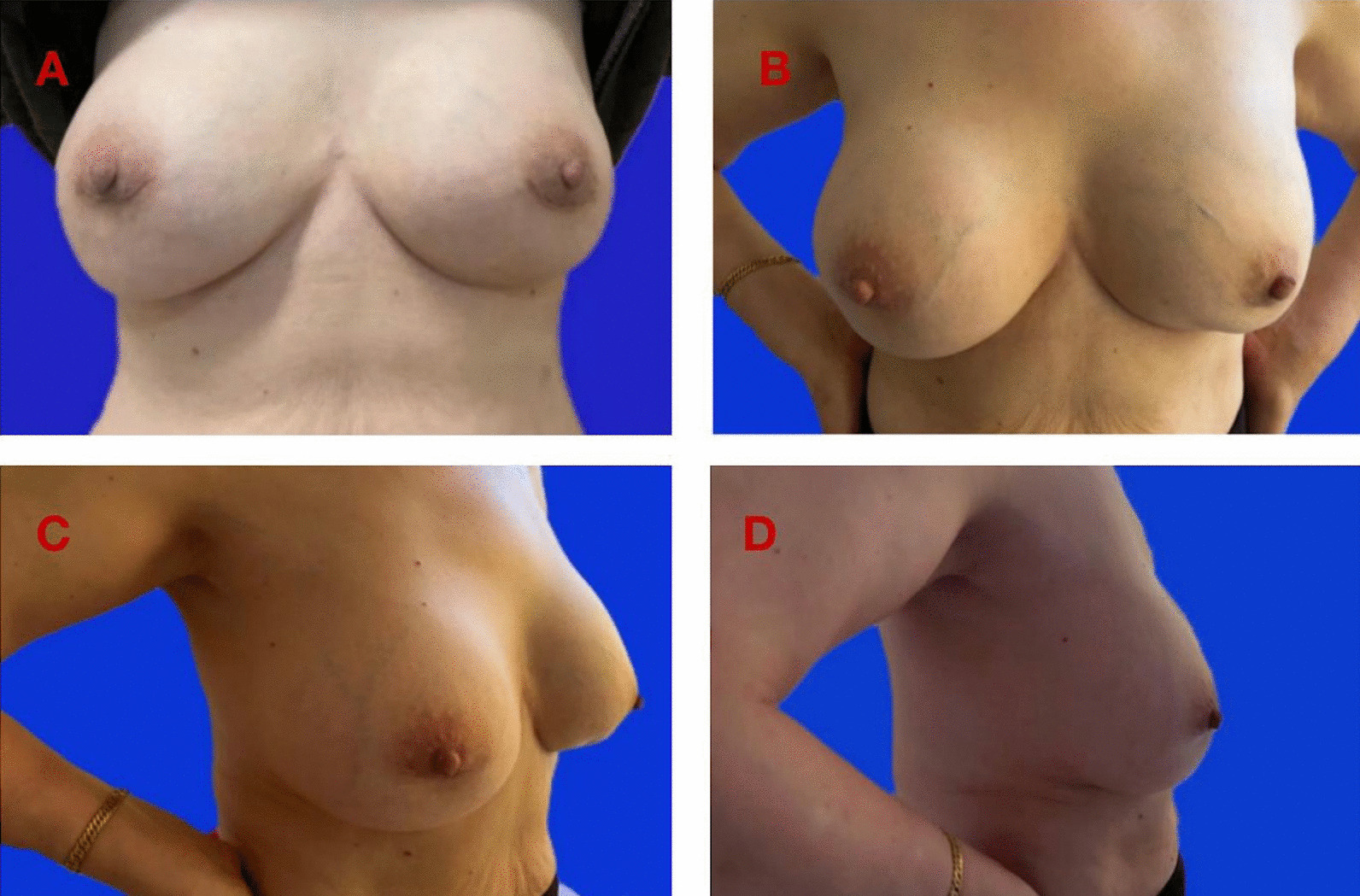
Cosmetic outcomes after immediate prosthetic breast reconstruction. A 67 years old woman was performed with immediate prosthetic breast reconstruction after PAAG removal (2 weeks after surgery). The size of the prostheses was 245 cc. **A** A photo at 2 months after surgery; **B**–**D** Three photos from three different angles at 2 years after surgery

Most of the reshaped breasts were natural in appearance (Fig. 2). Cosmetic outcomes were assessed by patients and the surgeon independently. The overall satisfaction rates by surgeon and patients were 88% and 92%, respectively (Table 3). The main concern of unsatisfied patients was the asymmetry of the nipples or breasts.

**Table 3:** Cosmetic results in the immediate breast reconstructed group

Assessor	Excellent	Good	Fair	Poor	Total	Satisfaction rate
Patients opinion	19	4	2	0	25	92%
Expert panel	18	4	3	0	25	88%

The BREAST-Q questionnaire was used to compared the HRQOL and patient satisfaction between the two groups. Seventeen patients in the immediate breast reconstructed group and fifty patients in the other group completed the BREAST-Q questionnaire by telephone follow-up recently, reflecting a 77.0 percent response rate. The mean follow-up time between the date of surgery and BREAST-Q completion was 42 ± 15 months (range 19–82). The BREAST-Q scores revealed that patients who accepted immediate prosthesis implantation had significant better outcomes in psychosocial well-being (*p* = 0.030), satisfaction with breasts (*p* = 0.021) (Table 4). But there were no statistic differences in physical well-being chest (*p* = 0.124), sexual well-being (*p* = 0.081), satisfaction with outcomes (*p* = 0.068), and satisfaction with care (*p* = 0.077) between the two groups. Besides, no capsular contraction was reported in the immediate breast reconstructed group during telephone follow-up.

**Table 4:** BREAST-Q scores comparing PAAG removal with or without immediate prosthetic breast reconstruction

BREAST-Q Module	PAAG removal with immediate prosthetic breast reconstruction*	PAAG removal without immediate prosthetic breast reconstruction*	*p* value	
Psychosocial well-being	28.9 ± 6.1	25.4 ± 1.9	0.030	
Sexual well-being	34.9 ± 4.1	33.0 ± 2.5	0.081	
Physical well-being chest	2.1 ± 3.3	3.6 ± 4.3	0.124	
Satisfaction with breasts	66.2 ± 12.6	58.4 ± 3.4	0.021	
Satisfaction with outcomes	72.1 ± 22.3	83.4 ± 13.8	0.068	
Satisfaction with care	75.4 ± 13.1	69.2 ± 6.7	0.077	

*Values are mean ± SD

## Discussion

Though CFDA has banned PAAG injection for more than 10 years, complications of PAAG injection are still prevalent and have become a major public health issue. The complications stimulate an increasing demand for PAAG removal. Surgeons have to carry out an extensive debridement to clear PAAG tissue as thoroughly as possible in most cases. However, an extensive surgery usually results in severe breast defect or deformity, which might leads to some negative impact on the patients’ physical and psychological health. Breast reconstruction is usually in demand to solve the aesthetic problem after PAAG removal. The ways of breast reconstruction include prosthesis implantation, autologous tissue breast reconstruction, and periareolar mammoplasty with the tissue folding technique (PMTFT) [11].

Silicone prosthesis might be an ideal implant to reshape the breasts after PAAG removal. Several studies suggested that prosthesis might be a useful material for plastic surgery after PAAG removal, and they even proposed flow diagrams to manage patients with complications after PAAG injection [10, 12, 18]. We performed immediate prosthetic breast reconstruction after PAAG removal through a small areolar incision assisted with an endoscope in selected cases. The results showed that this technique was feasible and safe, and the cosmetic outcomes were satisfactory. The BREASR-Q scores showed that immediate prosthesis implantation after PAAG removal was beneficial to improve patients’ psychosocial well-being and satisfaction with breasts.

Hence, it was suggested that this technique could be used as an alternative surgical option for patients with complications after PAAG injection. The advantages of this technique were described as follows:

1. Immediate breast reconstruction had some advantages when compared to delayed breast reconstruction. On one side, it relieved the physical trauma and economic burden due to a second operation. On the other side, it helped to reduce the psychic trauma resulted from breast defect after PAAG removal. It might be an appropriate surgical option if the inframammary fold and pectoral muscle were well protected, and PAAG tissue was cleared as thoroughly as possible.
2. The prostheses were implanted in the pre-pectoral plane, in other words, in the residual cavity after PAAG removal for several reasons. First of all, satisfactory aesthetic results could be achieved through pre-pectoral prosthetic breast reconstruction which was gaining popularity in the plastic surgery community [19]. Secondary, the soft breast tissue might be regarded as a natural and free “acellular dermal matrices (ADM)” to cover the prostheses. Thirdly, this procedure could relieve muscle spasms and animation deformity, which were common in the traditional prosthesis placement under the pectoralis major muscle [20].
3. It was more advantageous to remove PAAG tissue assisted with endoscopy. The endoscopic procedures allowed for better surgical field than direct visualization surgery, favouring complete PAAG removal and hemostasis. Moreover, it was an easier procedure for surgeons when compared to a total endoscopic surgery.
4. A small areolar incision was chosen to clear PAAG tissue for two reasons. In one part, the periareolar approach was valuable to remove PAAG to the greatest extent [21]. In another aspect, a small areolar incision was more private and less invasive.

It was worth noting that not all patients were suitable for immediate prosthetic breast reconstruction after PAAG removal. The indications, as we mentioned before, should be strictly controlled. The contraindications were as follows: (1) Obvious inflammation in the breasts, (2) Severe damage of the pectoralis major muscle or mammary gland, (3) Excess PAAG tissue remained, which could not be removed thoroughly, (4) Patients who had no desire of breast reconstruction. For the above cases, if the patients wished, delayed breast plastic surgery, such as prosthesis implantation, fat transplantation, etc., might be an option after the conditions of the breast improved.

There were two major limitations in this study. On one hand, partial follow-up of the patients was accomplished by telephone, because of the inconvenience of coming to the hospital. On the other hand, the questionnaire-based data of BREAST-Q were weakened to some extent by recall bias, inaccurate responses, and conversion to socially desirable responses. A closer follow-up was recommended in the future.

## Conclusion

Immediate prosthetic breast reconstruction after PAAG removal through a small areolar incision assisted with an endoscope may be a viable and safe technique with better psychosocial well-being and satisfaction with breasts. It may be an ideal surgical option for selected patients desiring a better breast appearance after PAAG removal. A larger cohort study is required in the future.

## Data Availability

The datasets of the current study are available from the corresponding author upon reasonable request.
